# Supplementary material for: Identification of a Quinone Derivative as a YAP/TEAD Activity Modulator from a Repurposing Library
Source: Pharmaceutics. 2022 Feb 10;14(2):391. doi: 10.3390/pharmaceutics14020391 (PMC8878929; doi:10.3390/pharmaceutics14020391)
Supplement: Supplementary file 1 [file pharmaceutics-14-00391-s001.zip › Supplementary Materials-checked.pdf]

pharmaceutics

Supplementary Materials:

Identification of a quinone derivative as YAP/TEAD activity modulator from a repurposing library

Angela Lauriola, Elisa Uliassi, Matteo Santucci, Maria Laura Bolognesi, Marco Mor, Laura Scalvini, Gian Marco Elisi, Gaia Gozzi, Lorenzo Tagliazucchi, Gaetano Marverti, Stefania Ferrari, Lorena Losi, Domenico D'Arca and Maria Paola Costi

Content Table:

Description of the Sunburst chart of the Hippo pathway inhibitors	S1
Biochemical connection of the chemical library to YAP-TEAD or to the Hippo pathway components.	S2
Chemistry Synthesis of ST11cis	S5
NMR spectra of DA15	S6-7
Table S1. List of the chemical library of 27 compounds tested in HaCat cells	S8
Table S2: Drug-likeness properties of compound library	S11
Table S3: Data summary from the PCA analysis	S12
Table S4. MTT cell growth inhibition (48h)	S13
Table S5. MTT cell growth inhibition (18h)	
Figure S1. Sunburst chart of the Hippo pathway inhibitors.	S14
Figure S2. In silico analysis for physico and chemical property characterization	S15
Figure S3. Scree-plot and variance explained for each principal component (PC)	S16
Figure S4. Growth inhibition assay	S17-19
Figure S5. MTT assay of HaCaT cells	S20
References	S21

Description of the Sunburst-chart of the current Hippo pathway direct/indirect regulators.

The schematic representation reports a summary of the most important current molecules able to block YAP/TEAD activity, both in terms of re-tasking for approved or investigational drugs and of under development small-molecule inhibitors. It is possible to distinguish two main macro-classes of YAP/TAZ-TEAD inhibitors: direct inhibitors, acting on the terminal effectors of Hippo signaling (YAP/TAZ; TEADs and YAP/TEAD complex) and indirect inhibitors acting as enhancers of Lats1/2-dependent YAP-phosphorylation. *Verteporfin, YAP-TAZ inhibitor 1* (tricyclic carbazole derivative) and *Celastrol* (pentacyclic terpenoid) [1] are YAP-TEAD dimer disruptors able to inhibit binding of YAP to TEAD and showing a downregulation of TEAD-responsive genes transcription in luciferase reporter gene assay. *Super-TDU, YAP-TEAD-IN-1, TB1G1* and *CDP3.1* are always YAP-TEAD dimer disruptors but targeting the TEAD's surface: in particular, *Super-TDU* is a Vgll4-mimicking peptide [2], *YAP-TEAD-IN-1* is a potent and competitive 17-mer cyclic-peptide inhibitor of YAP-TEAD interaction (IC_{50}=25nM), showing a higher binding affinity to TEAD1 (K_d=15nM) than YAP (K_d=40nM) [3], *TB1G1* is a TEAD-binding Cys-rich peptide (*optide*) sharing a *"LXXLF"* motif with YAP [4] and *CPD3.1* is a small-molecule inhibitor designed to target the TEAD surface interacting with

the YAP Ω-loop domain [5]. *All these compounds are* able to interrupt YAP/TEAD dimerization, showing a potent anti-tumor activity. Another important compound-class includes allosteric inhibitors occupying the palmitate-binding pocket (*PBP*) on TEAD-target protein: *TED-347, DC-TEADin02, K-975* and the *flufenamic acid derivatives*. They are all small-molecules able to inhibit the TEAD's transcriptional activity, but they are not disruptors of the YAP-TEAD complex. In particular, *TED-347* is a phenyl-amino-phenyl derivative inhibiting in irreversible, covalent and allosteric manner TEAD4-YAP1 complex (EC_{50} = 5.9 μM), by specifically binding with C367 residue within central pocket of TEAD4 (K_i = 10.3μM) [6]; *DC-TEADin02* is a naphthalene-sulfonamide derivative which is a potent and selective TEAD autopalmitoylation inhibitor [7] and, K-975 is a small-molecule with a strong inhibition effect against PPIs between YAP1-TEAD, acting at palmitate-binding-pocket of TEAD [8]. Moreover, there is also a wide range of drugs already in the clinic able to restrict YAP/TAZ-TEAD activities by cross-talking with their upstream regulators. To this aim, molecules acting on *GPCRs* (Glucagon, Epinephrin, Losartan and Dobutamine) and *VEGFR/EGFR cellular surface receptors* (Erlotinib, Pazopanib and Axitinib) attenuate YAP/TAZ-TEAD activity by enhancing the Lats1/2-dependent inhibitory YAP-phosphorylation; the *enhancers of cyclic AMP levels* promoting YAP/TAZ inhibitory phosphorylation (Forskolin, as adenylyl cyclase activator and, Theophylline as inhibitor of cyclic nucleotide phosphodiesterase); the *inhibitors of intracellular kinases and phosphatases* (Trametinib, Dasatinib and Calyculin-A) as well as the *inhibitors of mevalonate pathway* (Statins, Zoledronic acid and GGTI-298) inactivate YAP by preventing its nuclear entry and by shifting YAP/TAZ to the cytoplasm; epigenetic modulators, especially *HDAC-* and *BET-inhibitors* (Panobinostat, Vormostat, I-BET151 and JQ1) transcriptionally repress the expression of YAP leading to a decrease of YAP levels; *AMPK/PKA interplay modulators* (Metformin and AICAR) enhance YAP/TAZ inhibitory phosphorylation, cytoplasmic retention and suppress the YAP-TAZ mediated transcription by activating AMPK, which in turn phosphorylates and stabilizes AMOT protein, a negative regulator of YAP/TAZ; the cytoskeletal actin/myosin modulators, (*Latrunculin-A, Blebbistatin, Cytochalasin-D, XAV-939 and ML-7*) have an indirect inhibitory effect on YAP/TAZ by inhibiting the actin polymerization. *ML-7* compound is a naphthalene-sulfonamide small-molecule derivative active against Myosin Light Chain Kinase, MLCK (IC_{50} = 300nM), which also inhibits YAP/TAZ protein [9] and, interestingly, *Cytochalasin-D* and *XAV-939* are both able to suppress the YAP-TEAD transcriptional activity by interfering with cytoskeletal-actin polymerization and, this latter is also able to act as a Tankyrase kinase inhibitor (TNKS) aiming to maintain the stabilization of AMOT and to promote YAP cytoplasmic-retention [10].

Biochemical connection of the chemical library to YAP-TEAD or to the Hippo pathway components.

Our in-house libraries were visually inspected to select lead-like compounds based on: (i) chemical diversity, (ii) potential pathway connection to YAP-TEAD activity, and (iii) features suitable for PPI inhibition (PPIi). Thus, main PPIi privileged chemotypes have been identified to guarantee a high degree of structural diversity of the library (Figure 2) and a final set of 27 compounds was assembled for cell-based screening assay (Table S1) on the basis of potential pathway connection to YAP-TEAD activity.

In detail, the quinone scaffold (memoquin, **DA15**, **SVT118**, **IA5**, **EU11**) can be considered a truly privileged motif for interfering with protein–protein interactions due to the ability to establish hydrogen bond with the carbonyl groups and π-stacking interactions through the planar core [11]. Moreover, the quinones bearing a polyamine backbone have the additional chance to recognize multiple anionic sites of the target by assuming different

protonated conformations [11]. Furthermore, the regenerative potential of vitamin K homologues has been recently disclosed. In particular, vitamin K derivatives have been shown to selectively modulate the differentiation of neuronal progenitor cells into neurons [12]. Quinazoline and quinolines (**CDM38**, **BAT33**, **UPR1268**) also represent a privileged structure and a good template for lead generation library. Indeed, numerous quinazoline-based derivatives with different biological actions have been successfully introduced to the market (e.g., the sympatholytic drug Prazosin and the anticancer drugs Gefitinib and Erlotinib). Notably, the epidermal growth factor receptor (EGFR) inhibitor Erlotinib has been reported to increase mobilization of hematopoietic stem and progenitor cells [13]. Interestingly, quinolinols were identified as YAP/TAZ–TEAD activity modulators, being able to bind the palmitate-binding pocket (PBP) of TEAD [14]. In addition, *N*-aryl sulphonamide-quinazoline derivatives were demonstrated potential anti-gastric cancer agents via modulation of the Hippo signaling pathway [15]. It also should be noted that quinazoline **BAT33** might have the possibility to interact covalently with the target through the disulfide bridge, conferring a reversible covalent inhibition.

Another interesting class of compounds is that of stilbene-based molecules (**ST11** cis/trans), which have been selected because of their multiple beneficial activities against different molecular targets. Remarkably, resveratrol, the most studied among the stilbene derivatives, has shown to suppress breast cancer cell invasion by inactivating a RhoA/YAP signaling axis [16]. In addition, the selected stilbene derivatives might be useful to explore the putative stereochemical preference of the target (if any) between the trans and cis isomers. Regarding heterocyclic compounds -thiazolidindione (**ACG35**), diketopiperazine (**JCM24**), tetrahydroacridine (**RS34**), dibenzodiazepinone (**ADC3**); carbazole (**ULA26**), the selection spans a relatively wide range of scaffolds present in our in-house library. Particularly, thiazolidindione derivatives **ACG35**, developed as GSK-3 structural motif, featured the core scaffold of PPARγ agonists antihyperglycemic drugs. Such PPARγ agonists promoted differentiation of cancer stem cells by modulating YAP transcriptional activity [17]. The bivalent tetrahydroacridine **RS34** is a close analogue of bis(propyl)-cognitin, which exhibits neurite outgrowth-promoting activities [18]. Conversely, the tryciclic pyridobenzodiazepinone scaffold of **ADC3** is present in the structure of the M1 selective antagonist, pirenzepine used in the treatment of ulcers. Nevertheless, it has been shown that M1 receptor has been considered a potential target to modulate oligodendrocyte progenitor survival, proliferation, and differentiation [19]. We also included the carbazole derivative **ULA26**, properly designed for the neuroregenerative properties [20].

Steroids (pristimerin, **PCM129, UPR1355**, digoxin, deslanoside) is another interesting class included in our library. Digoxin and Deslanoside which should be able to mimic interactions of YAP physiological inhibitors at level of WW-domain on the YAP protein, have been included. For this reason, they do not act at YAP-TEAD complex interface but they should function as YAP-targeting molecules modifying YAP molecular structure, preventing YAP-TEAD interaction and then inhibiting YAP activity function. Furthermore, steroid hormones glucocorticoids act as hormonal modulator of YAP [21].

Carbamates (**URB694**, **URB913**) and anilino-alkyl-amides (**UCM765**, **UCM871**) are inhibitors of endocannabinoids degrading enzymes: an agonist of MT1 receptor; a partial agonist of MT2 receptor; an EGFR/HER2 inhibitor and a PPAR-α agonist, whose cross-talks with the Hippo pathway have been highlighted [22,23].

Chemistry

Synthesis of (z)-2-(3,5-dimethoxystyryl)phenol (ST11cis)

Compound ST11cis was obtained taking advantage of a standard Wittig reaction between the 2-(tert-butyldimethylsilyloxy)benzaldehyde and the ylide obtained by the treatment of (3,5-dimethoxybenzyl)triphenylphosphonium bromide with n-butillithium as previously described [24]. General methods for silyloxy protection and deprotection were followed.^{1}H-NMR (300 Mz, $CDCl_3$) δ 7.25-7.18 (m, 2H), 6.94-6.89 (m, 2H), 6.73 (d, J = 12.0 Hz, 1H), 6.63 (d, J = 12.0 Hz, 1H), 6.43-6.36 (m, 3H), 5.36 (s, br, 1H), 3.62 (s, 6H). ^{13}C-NMR (75 MHz, $CDCl_3$) δ 160.5, 152.5, 137.7, 132.8, 129.6, 129.0, 124.9, 123.9, 120.5, 115.7, 106.4, 100.7, 55.0. IR (CCl_4) cm^{-1} 3549, 3003, 2935, 2837, 1591, 1487, 1461, 1427, 1336, 1302, 1262, 1207, 1196, 1157, 1068. ESI-MS for $C_{16}H_{16}O_3$: calculated 256.11, found m/z 257.22 [M + H]$^+$. UPLC-MS purity (UV at 254 nm) 98%.

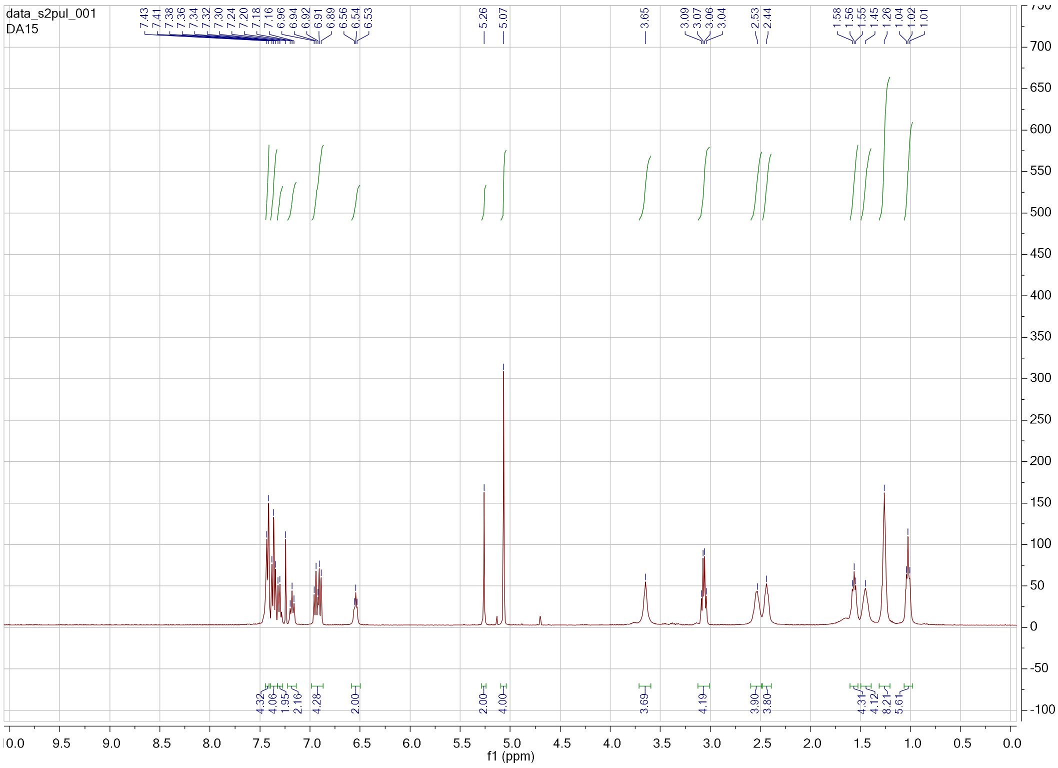

^{1}H-NMR (400 Mz, $CDCl_3$) of **DA15**

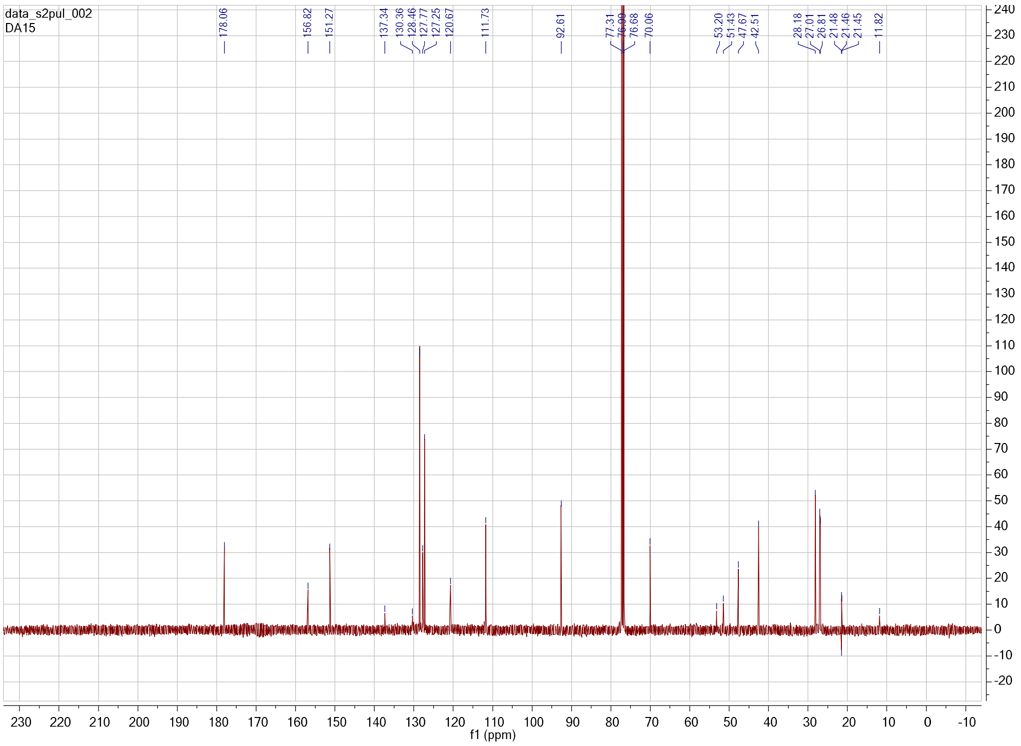

^{13}C-NMR (100 Mz, $CDCl_3$) of **DA15**

Table S1. Chemical structure of the 27 compounds of library tested in HaCat cells and reference reporting their biological mechanism justifying their inclusion in the library.

COMPOUND	TARGET	STRUCTURE	REF.
**ACG35**	GSK-3β	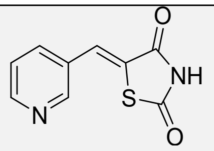	[25]
**JCM24**	PrP[sc] aggregation	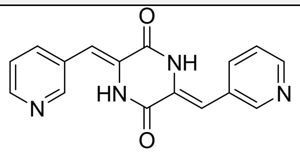	[26]
**EU11**	*T. cruzi* trypanothione reductase	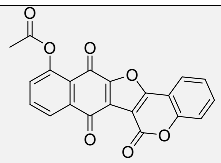	[27]
**MEMOQUIN**	Aβ aggregation, *h*AChE, BACE-1	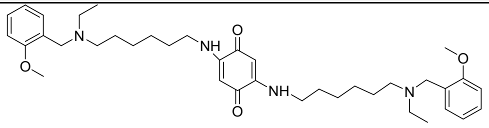	[28]
**DA15**	-	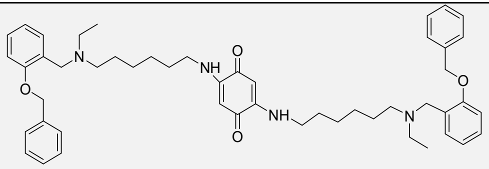	n.p.
**SVT118**	PrP[sc] aggregation	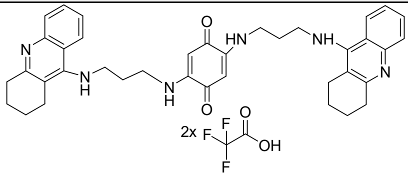	[29]
**RS34**	Aβ aggregation, *h*AChE	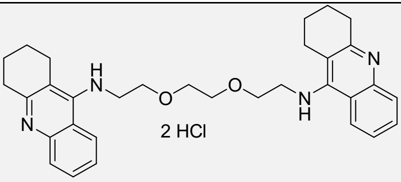	[30]
**ANTIPAO**	polyamine oxidase	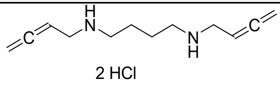	[31]
**CDM38**	Adrenergic receptors (α_{1A}, α_{1B}, α_{1D})	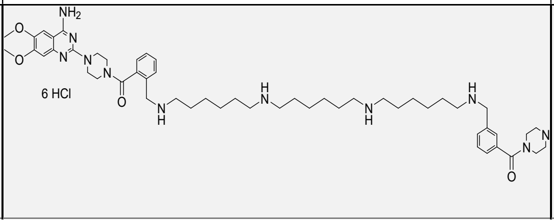	[32]

**BAT33**	Adrenergic receptors (α_{1A}, α_{1B}, α_{1D}, α_2)	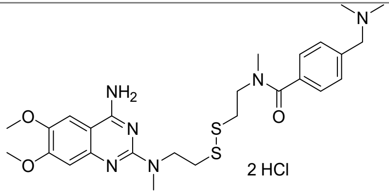	[33]
**ULA26**	Neuroregenerative Phenotypic hit (CGNs, HepG2)	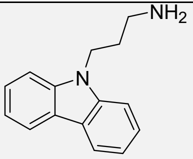	[21]
**IA5**	Anti-*T. cruzi* Phenotypic hit	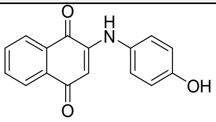	[34]
**ADC3**	-	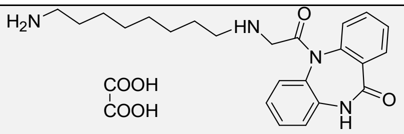	n.p.
**ST11TRANS**	Anticancer Phenotypic hit (HL60 and K562 cells)	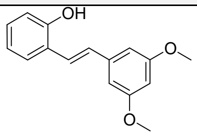	[24]
**ST11CIS**	-	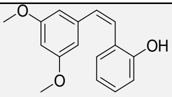	n.p.
**URB694**	FAAH	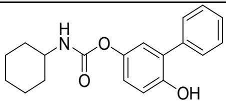	[35]
**UPR1260.0 0/1201**	MGL (ox Cys)	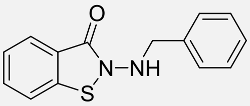	n.p
**PRISTIMERIN (UPR1265.0 0/1301)**	MGL (allosteric-modulator), HSP90, NFkB	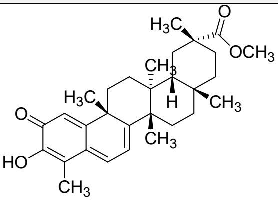	[36]
**UCM871**	Agonist MT1	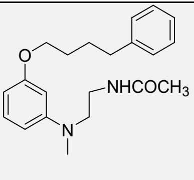	[37]

**UCM765**	Partial agonist MT2	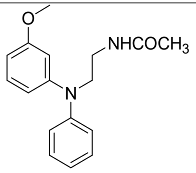	[38]
**UPR1268.0 0/1203**	Inhibitor EGFR/HER2	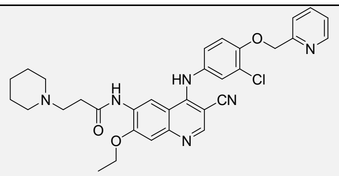	[39]
**URB913**	Inhibitor NAAA	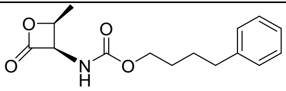	[40]
**UPR1320.0 0/1302**	PPAR-α agonist	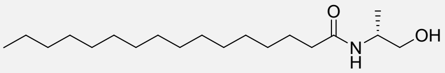	n.p.
**PCM129**	Ephrine antagonist (receptor EphA2)	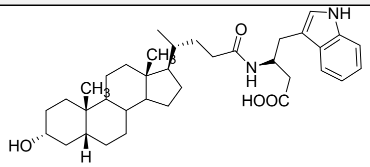	[41]
**UPR1355.0 0/1301**	Ephrine antagonist (receptor EphA2)	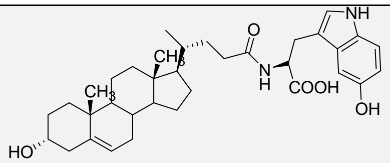	[42]
**DIGOXIN**	Na^+/K^+ ATPase	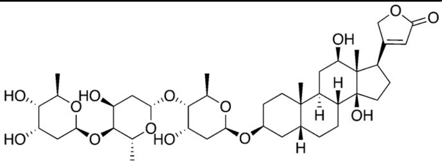	[43]
**DESLANOSIDE**	Na^+/K^+ ATPase	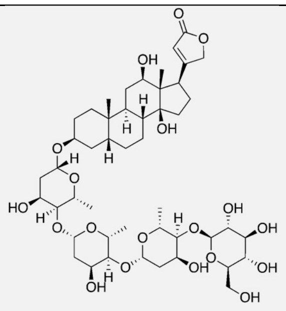	[43]

Table S2: Drug-likeness properties of compound library.

Physiochemical Property	Minimum Value	Maximum Value	Average	Drug-likeness criteria	% compounds according to RO5
**MW (g/mol)**	206	1344	491	≤500	58.6%
**Alog P**	1.18	9.05	4.09	≤5	62.1%
**HBA**	0	19	4.6	≤10	93.1%
**HBD**	0	9	2.0	≤5	93.1%
**Total Polar Surface Area (Å²)**	24	282	100.6	≤140	75.9%
**N° of Rotable Bonds**	1	28	8.4	≤10	65.5%
* % of compounds according to RO5 (cut-off selection: no more than one violation per compound)					51.7%

Table S3: Data summary with relevant parameters for principal components from the PCA analysis.

	**PC1**	**PC2**	**PC3**	**PC4**	**PC5**	**PC6**
**Eigenvalue**	3,380235	1,765045	0,348073	0,230192	0,13718	0,039274
**Cumulative Variance**	0,785378	0,987388	0,995244	0,99868	0,9999	1
**Variance Explained**	0,572921	0,29916	0,058996	0,039016	0,023251	0,006657
**Loading value_AlogP**	0,1346	0,6661	-0,5682	0,4144	0,0443	0,2043
**Loading value _HBA**	0,4560	-0,3132	0,0617	0,3009	0,7656	0,1159
**Loading value _HBD**	0,4136	-0,3258	-0,6661	-0,5132	-0,1250	0,0051
**Loading value _MW**	0,5053	0,2020	0,1445	0,1215	-0,1564	-0,8023
**Loading value _TPSA**	0,4928	-0,1738	0,2646	0,3101	-0,5796	0,4742
**Loading value _RBs**	0,3234	0,5302	0,3725	-0,6029	0,1894	0,2760

Table S4. MTT cell growth inhibition of the selected compounds after 48h against HaCat cells. Mean±SEM (n = 3).

COMPOUND	IC_{50} (µM) (48H)
VERTEPORFIN	5.0 ± 0.3
MEMOQUIN	7.2 ± 0.13
IA5	7.3 ± 1.43
UPR1268	1.7 ± 0.11
DA15	4.0 ± 0.56
PRISTIMERIN	0.8 ± 0.17
DIGOXIN	0.2 ± 0.05
DESLANOSIDE	0.25 ± 0.1

Table S5. MTT growth inhibition assay in HaCaT cells at 18 hours for the 7 active compounds. ND indicates the not detectable IC50 at the highest concentration used. IC50 values are the mean of from three independent experiments (n = 3).

**IC_{50} (µM)-18 hours in HaCaT**	
VERTEPORFIN	ND
MEMOQUIN	ND
IA5	ND
UPR1268	3.2
DA15	3.4
PRISTIMERIN	1.64
DIGOXIN	0,5
DESLANOSIDE	0.7

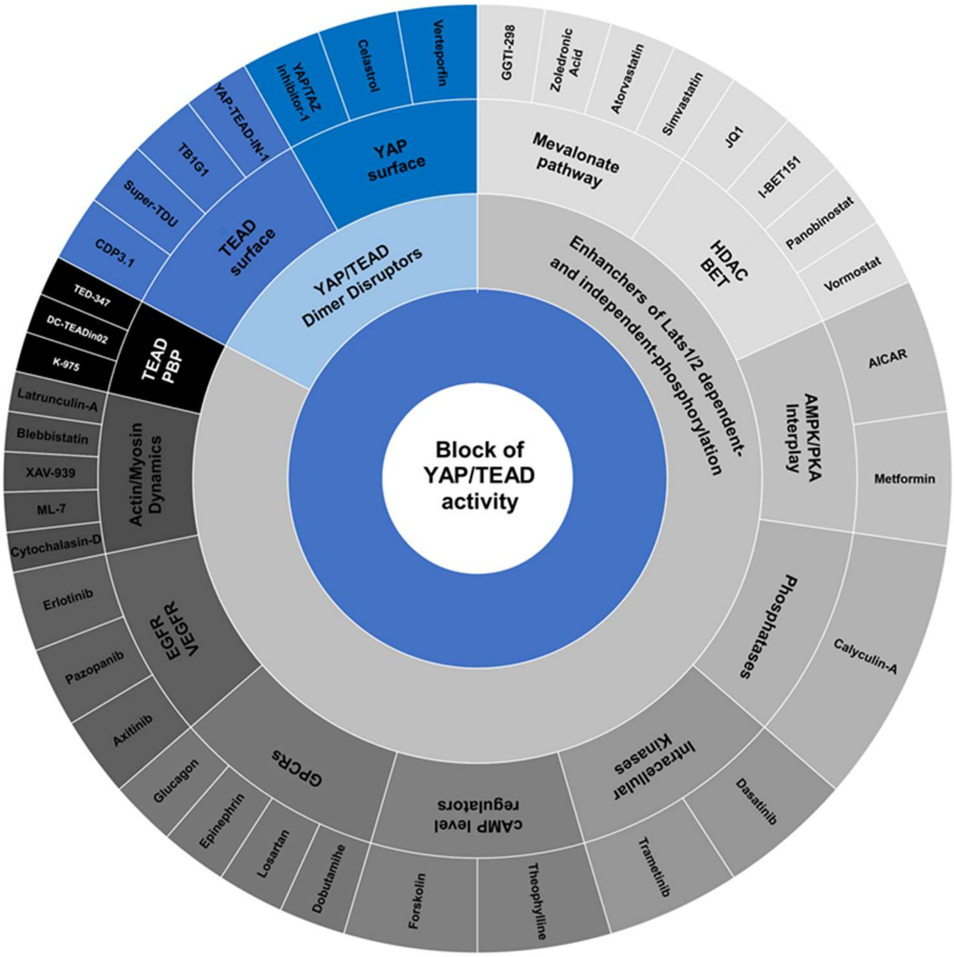

Figure S1. Sunburst chart of the Hippo pathway inhibitors. Schematic representation of the most important inhibitors able to block YAP/TEAD function activity, including both repurposing and under development compounds (experimental/investigational status). In particular, the outer ring of the graph reports the name of the compounds, the second ring shows the targeting pathway or protein, while in the middle ring the mechanism of action has been specified.

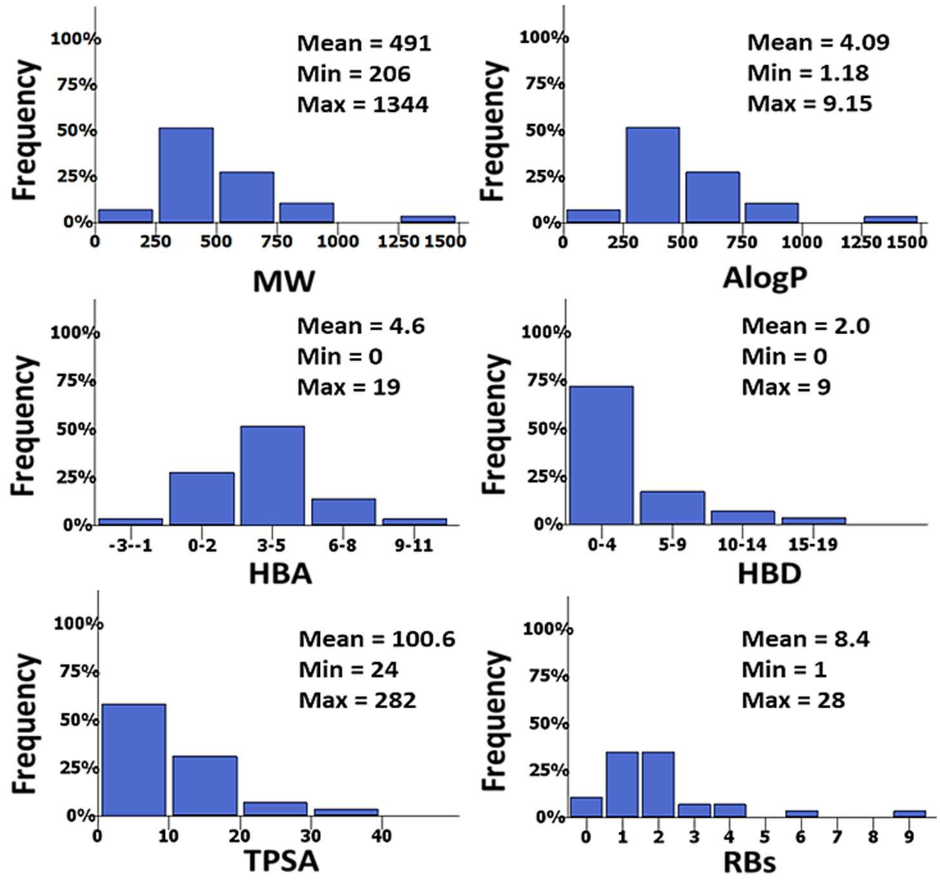

Figure S2. In silico analysis for physical and chemical property characterization. The figure shows the six molecular descriptors used to describe the physicochemical properties for each compound of the entire library dataset. For each molecular parameter, the distribution of computational values has been reported (percentage of frequency).

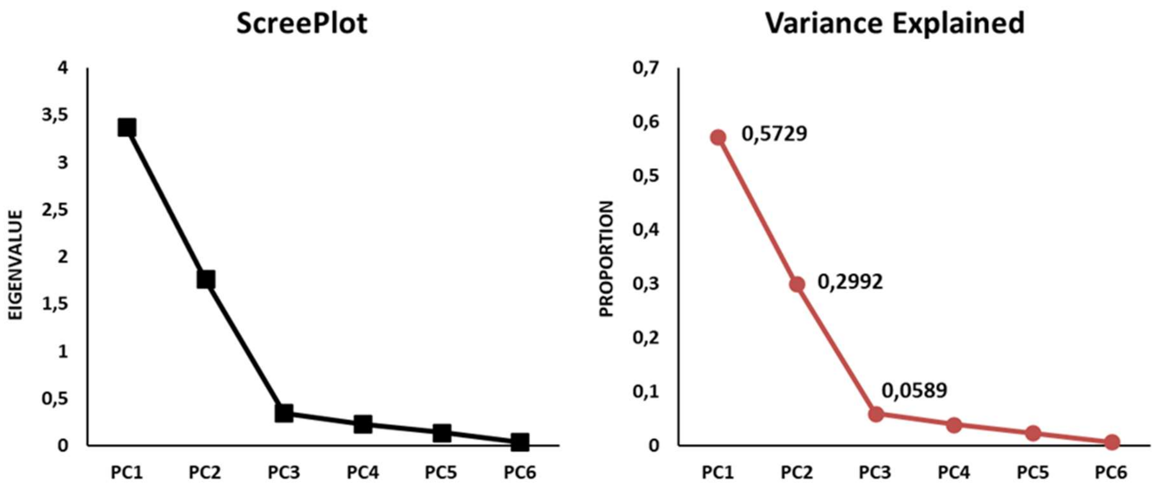

Figure S3. *Scree-plot and variance explained for each principal component (PC).* The graph shows a line-plot of the eigenvalues, calculated for each PC and ordered from largest to smallest (*left*) and the respective amount of variance explained by each component has been also calculated (*right*). The percentage of explained variance for the first three principal components PC1, PC2, PC3 is equal to 57.3%, 29.9% and 5.9%, respectively. Setting 80% as predetermined threshold, PC1 and PC2 cumulatively explain 87.2% of the total variance.

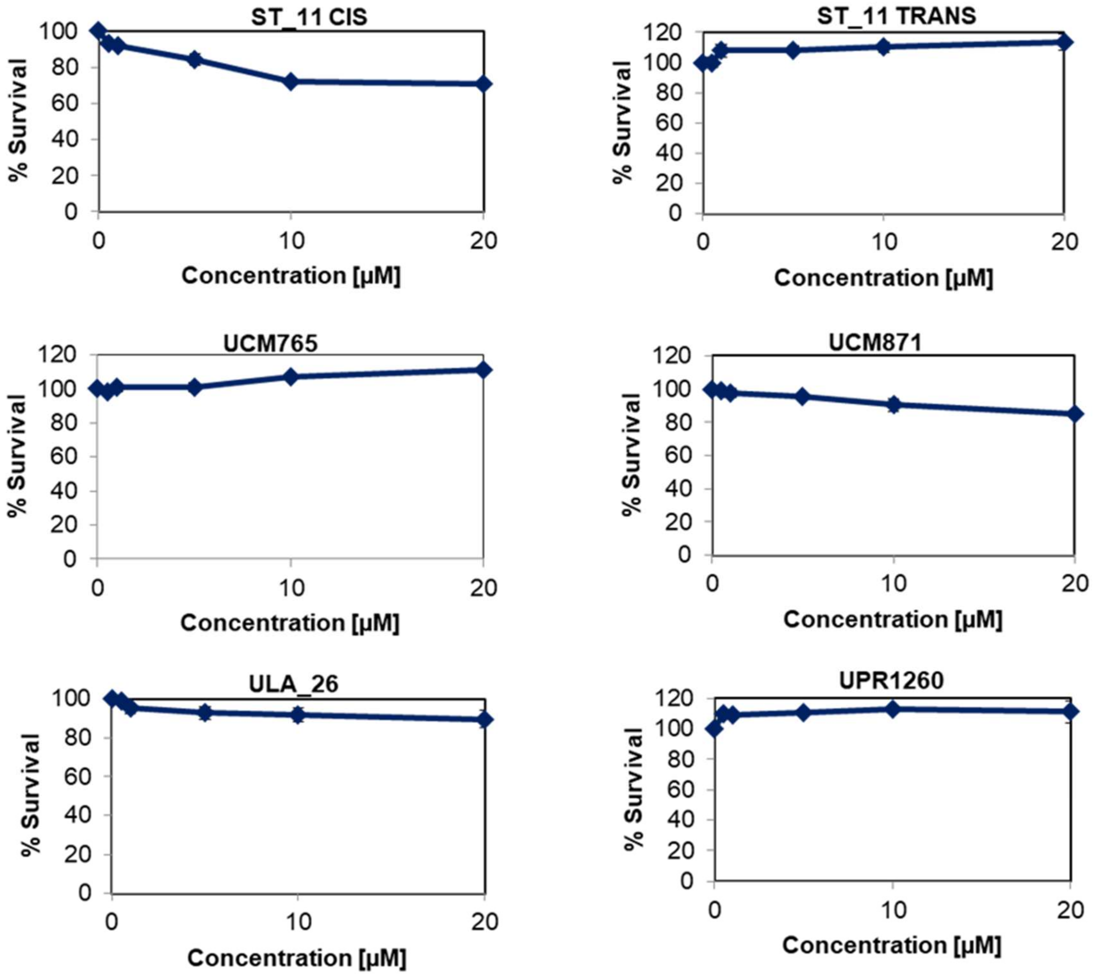

Figure S4 (a). Growth inhibition assay in HaCaT cells at 48 hours for all compounds tested excluding those showing a measurable IC_{50} and reported in the main text.

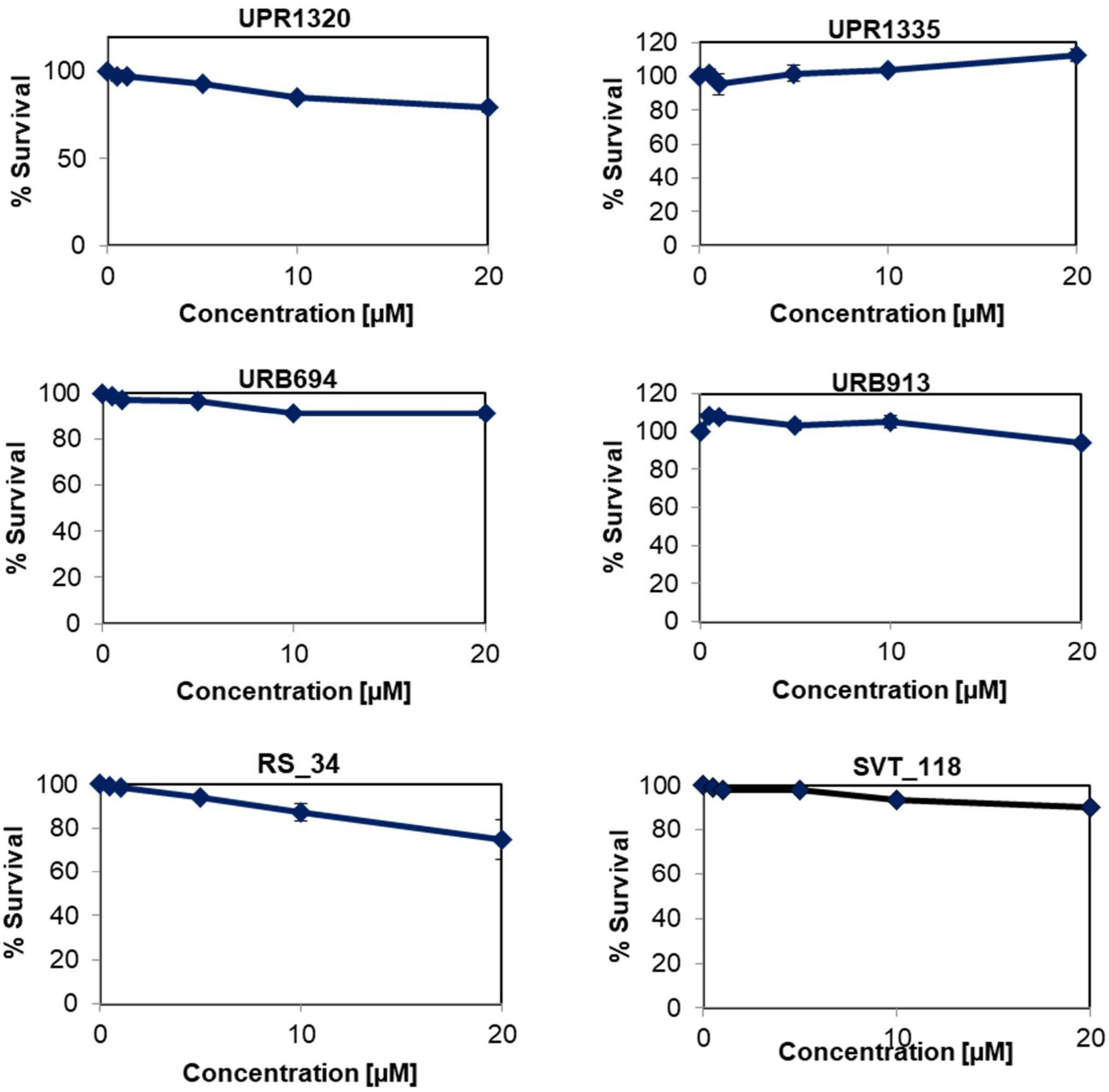

Figure S4(b). Growth inhibition assay in HaCaT cells at 48 hours for all compounds tested excluding those showing a measurable IC_{50} and reported in the main text.

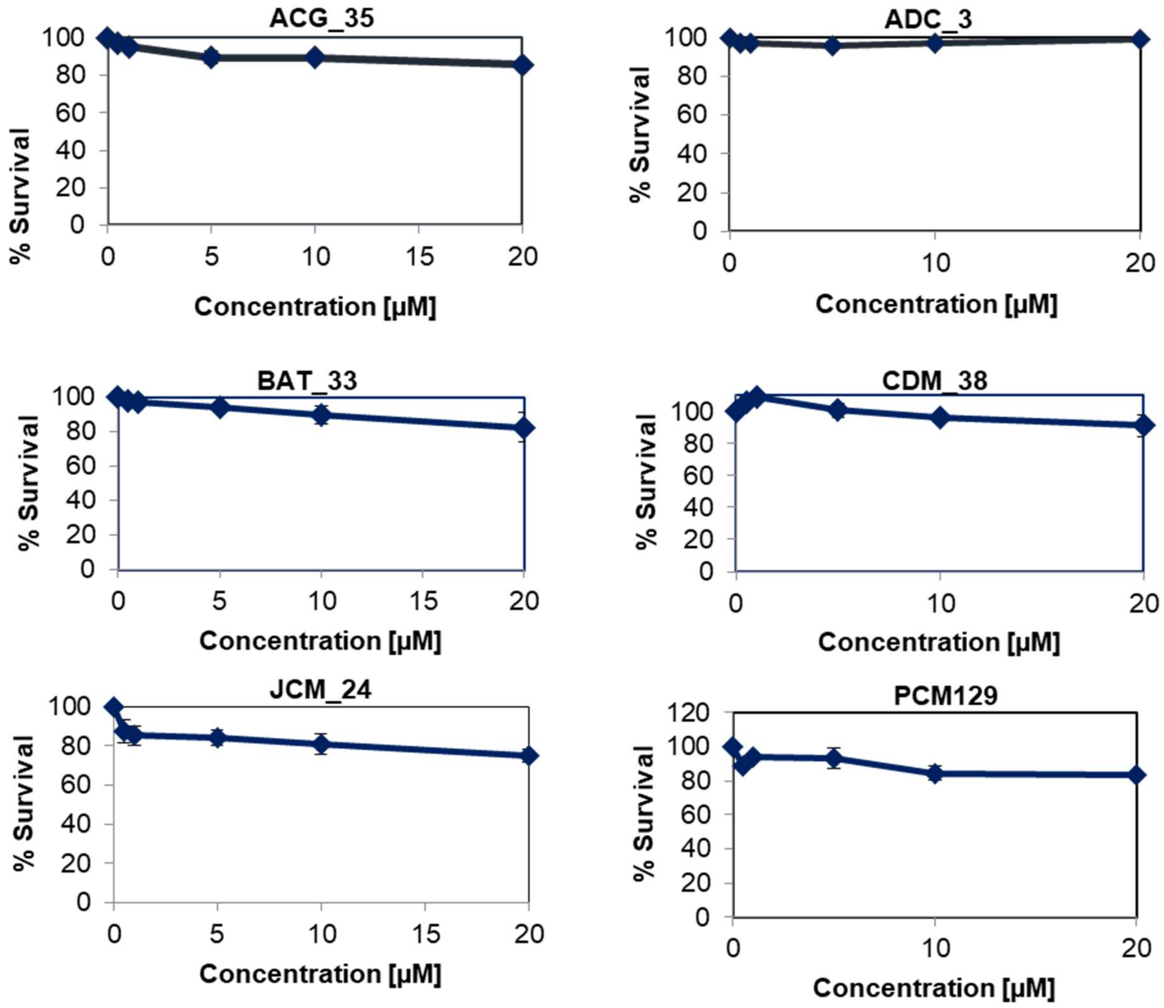

Figure S4(c). Growth inhibition assay in HaCaT cells at 48 hours for all compounds tested excluding those showing a measurable IC_{50} and reported in the main text.

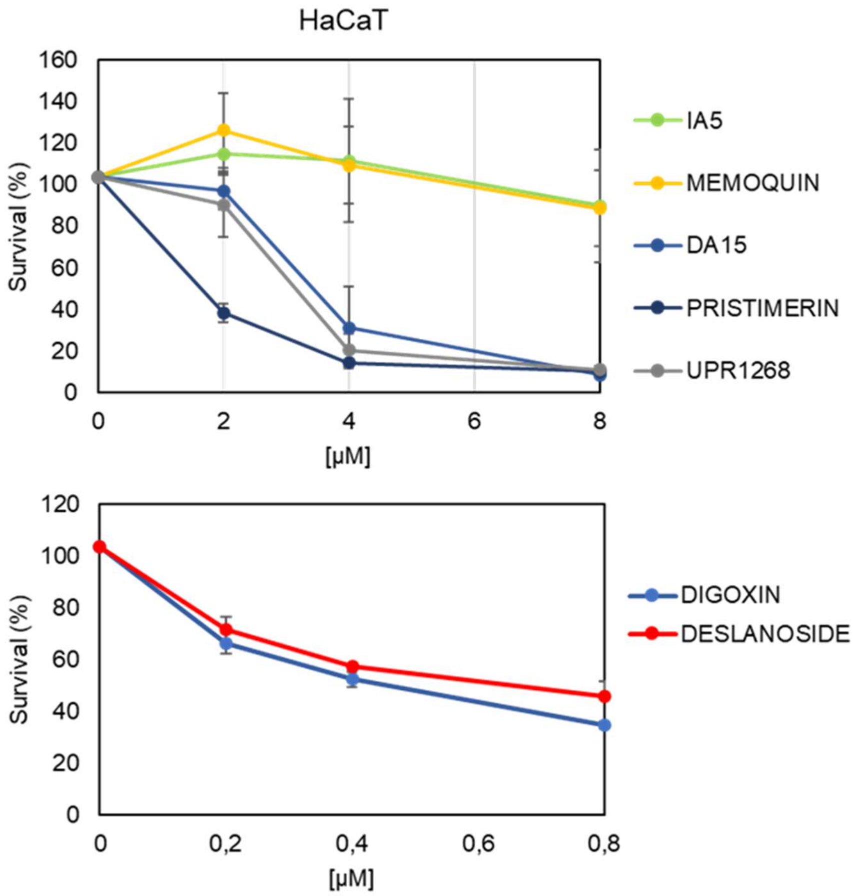

Figure S5. MTT assay of HaCaT cells treated with the selected compound for 18 hours. Error bars represent s.d. (n=3).

References

[1] K. Nouri, T. Azad, M. Ling, H.J.J. van Rensburg, A. Pipchuk, H. Shen, Y. Hao, J. Zhang, X. Yang, Identification of Celastrol as a Novel YAP-TEAD Inhibitor for Cancer Therapy by High Throughput Screening with Ultrasensitive YAP/TAZ–TEAD Biosensors, Cancers. 11 (2019) 1596. https://doi.org/10.3390/cancers11101596.

[2] S. Jiao, H. Wang, Z. Shi, A. Dong, W. Zhang, X. Song, F. He, Y. Wang, Z. Zhang, W. Wang, X. Wang, T. Guo, P. Li, Y. Zhao, H. Ji, L. Zhang, Z. Zhou, A Peptide Mimicking VGLL4 Function Acts as a YAP Antagonist Therapy against Gastric Cancer, Cancer Cell. 25 (2014) 166–180. https://doi.org/10.1016/j.ccr.2014.01.010.

[3] P. Furet, B. Salem, Y. Mesrouze, T. Schmelzle, I. Lewis, J. Kallen, P. Chène, Structure-based design of potent linear peptide inhibitors of the YAP-TEAD protein-protein interaction derived from the YAP omega-loop sequence, Bioorganic & Medicinal Chemistry Letters. 29 (2019) 2316–2319. https://doi.org/10.1016/j.bmcl.2019.06.022.

[4] Z.R. Crook, G.P. Sevilla, D. Friend, M.-Y. Brusniak, A.D. Bandaranayake, M. Clarke, M. Gewe, A.J. Mhyre, D. Baker, R.K. Strong, P. Bradley, J.M. Olson, Mammalian display screening of diverse cystine-dense peptides for difficult to drug targets, Nat Commun. 8 (2017) 2244. https://doi.org/10.1038/s41467-017-02098-8.

[5] S.A. Smith, R.B. Sessions, D.K. Shoemark, C. Williams, R. Ebrahimighaei, M.C. McNeill, M.P. Crump, T.R. McKay, G. Harris, A.C. Newby, M. Bond, Antiproliferative and Antimigratory Effects of a Novel YAP–TEAD Interaction Inhibitor Identified Using in Silico Molecular Docking, J. Med. Chem. 62 (2019) 1291–1305. https://doi.org/10.1021/acs.jmedchem.8b01402.

[6] K. Bum-Erdene, D. Zhou, G. Gonzalez-Gutierrez, M.K. Ghozayel, Y. Si, D. Xu, H.E. Shannon, B.J. Bailey, T.W. Corson, K.E. Pollok, C.D. Wells, S.O. Meroueh, Small-Molecule Covalent Modification of Conserved Cysteine Leads to Allosteric Inhibition of the TEAD·Yap Protein-Protein Interaction, Cell Chemical Biology. 26 (2019) 378-389.e13. https://doi.org/10.1016/j.chembiol.2018.11.010.

[7] W. Lu, J. Wang, Y. Li, H. Tao, H. Xiong, F. Lian, J. Gao, H. Ma, T. Lu, D. Zhang, X. Ye, H. Ding, L. Yue, Y. Zhang, H. Tang, N. Zhang, Y. Yang, H. Jiang, K. Chen, B. Zhou, C. Luo, Discovery and biological evaluation of vinylsulfonamide derivatives as

highly potent, covalent TEAD autopalmitoylation inhibitors, European Journal of Medicinal Chemistry. 184 (2019) 111767. https://doi.org/10.1016/j.ejmech.2019.111767.

[8] A. Kaneda, T. Seike, T. Danjo, T. Nakajima, N. Otsubo, D. Yamaguchi, Y. Tsuji, K. Hamaguchi, M. Yasunaga, Y. Nishiya, M. Suzuki, J.-I. Saito, R. Yatsunami, S. Nakamura, Y. Sekido, K. Mori, The novel potent TEAD inhibitor, K-975, inhibits YAP1/TAZ-TEAD protein-protein interactions and exerts an anti-tumor effect on malignant pleural mesothelioma, Am J Cancer Res. 10 (2020) 4399–4415.

[9] A.V. Pobbati, W. Hong, A combat with the YAP/TAZ-TEAD oncoproteins for cancer therapy, Theranostics. 10 (2020) 3622–3635. https://doi.org/10.7150/thno.40889.

[10] W. Wang, N. Li, X. Li, M.K. Tran, X. Han, J. Chen, Tankyrase Inhibitors Target YAP by Stabilizing Angiomotin Family Proteins, Cell Reports. 13 (2015) 524–532. https://doi.org/10.1016/j.celrep.2015.09.014.

[11] Prati, F.; Uliassi, E.; Bolognesi, M. L. Two diseases, one approach: multitarget drug discovery in Alzheimer's and neglected tropical diseases. MedChemComm**,** 5 (2014) 853-861. https://doi.org/10.1039/C4MD00069B.

[12] Kimura, K.; Hirota, Y.; Kuwahara, S.; Takeuchi, A.; Tode, C.; Wada, A.; Osakabe, N.; Suhara, Y. Synthesis of Novel Synthetic Vitamin K Analogues Prepared by Introduction of a Heteroatom and a Phenyl Group That Induce Highly Selective Neuronal Differentiation of Neuronal Progenitor Cells. J Med Chem 60(2017) 2591–2596. https://doi.org/10.1021/acs.jmedchem.6b01717.

[13] Ryan, M. A.; Nattamai, K. J.; Xing, E.; Schleimer, D.; Daria, D.; Sengupta, A.; Kohler, A.; Liu, W.; Gunzer, M.; Jansen, M.; Ratner, N.; Le Cras, T. D.; Waterstrat, A.; Van Zant, G.; Cancelas, J. A.; Zheng, Y.; Geiger, H. Pharmacological inhibition of EGFR signaling enhances G-CSF-induced hematopoietic stem cell mobilization. Nat Med**,** 16 (2010) 1141-6. doi: 10.1038/nm.2217.

[14] Pobbati AV, Mejuch T, Chakraborty S, Karatas H, Bharath SR, Guéret SM, Goy PA, Hahne G, Pahl A, Sievers S, Guccione E, Song H, Waldmann H, Hong W. Identification of Quinolinols as Activators of TEAD-Dependent Transcription. ACS Chem Biol. 14 (2019) 2909-2921. doi: 10.1021/acschembio.9b00786.

[15] Niu JB, Hua CQ, Liu Y, Yu GX, Yang JJ, Li YR, Zhang YB, Qi YQ, Song J, Jin CY, Zhang SY. Discovery of *N*-aryl sulphonamide-quinazoline derivatives as anti-gastric cancer agents *in vitro* and *in vivo via* activating the Hippo signalling pathway. J Enzyme Inhib Med Chem. 36 (2021) 1715-1731. doi: 10.1080/14756366.2021.1958211.

[16] Kim YN, Choe SR, Cho KH, Cho DY, Kang J, Park CG, Lee HY. Resveratrol suppresses breast cancer cell invasion by inactivating a RhoA/YAP signaling axis. Exp Mol Med. 49 (2017) e296. doi: 10.1038/emm.2016.151.

[17] Basu-Roy U, Han E, Rattanakorn K, Gadi A, Verma N, Maurizi G, Gunaratne PH, Coarfa C, Kennedy OD, Garabedian MJ, Basilico C, Mansukhani A. PPARγ agonists promote differentiation of cancer stem cells by restraining YAP transcriptional activity. Oncotarget. 7 (2016) 60954-60970. doi: 10.18632/oncotarget.11273.

[18] Hu, S.; Cui, W.; Mak, S.; Xu, D.; Hu, Y.; Tang, J.; Choi, C.; Lee, M.; Pang, Y.; Han, Y. Substantial Neuroprotective and Neurite Outgrowth-Promoting Activities by Bis(propyl)-cognitin via the Activation of Alpha7-nAChR, a Promising Anti-Alzheimer's Dimer. ACS Chem Neurosci, 6 (2015) 1536-45. https://doi.org/10.1021/acschemneuro.5b00108

[19] De Angelis, F.; Bernardo, A.; Magnaghi, V.; Minghetti, L.; Tata, A. M. Muscarinic receptor subtypes as potential targets to modulate oligodendrocyte progenitor survival, proliferation, and differentiation. Developmental Neurobiology**,** 72 (2012) 713-728. doi: 10.1002/dneu.20976.

[20] E. Uliassi, L.E. Peña-Altamira, A.V. Morales, F. Massenzio, S. Petralla, M. Rossi, M. Roberti, L. Martinez Gonzalez, A. Martinez, B. Monti, M.L. Bolognesi, A Focused Library of Psychotropic Analogues with Neuroprotective and Neuroregenerative Potential, ACS Chem. Neurosci. 10 (2019) 279–294. https://doi.org/10.1021/acschemneuro.8b00242.

[21] Sorrentino G, Ruggeri N, Zannini A, Ingallina E, Bertolio R, Marotta C, Neri C, Cappuzzello E, Forcato M, Rosato A, Mano M, Bicciato S, Del Sal G. Glucocorticoid receptor signalling activates YAP in breast cancer. Nat Commun. 8 (2017) 14073. doi: 10.1038/ncomms14073.

[22] Azad T, Rezaei R, Surendran A, Singaravelu R, Boulton S, Dave J, Bell JC, Ilkow CS. Hippo Signaling Pathway as a Central Mediator of Receptors Tyrosine Kinases (RTKs) in Tumorigenesis. Cancers (Basel). 12 (2020 :2042. doi: 10.3390/cancers12082042.

[23] Lo Sardo F, Muti P, Blandino G, Strano S. Melatonin and Hippo Pathway: Is There Existing Cross-Talk? Int J Mol Sci. 18 (2017), 1913. doi: 10.3390/ijms18091913.

[24] M. Roberti, D. Pizzirani, M. Recanatini, D. Simoni, S. Grimaudo, Di Cristina, V. Abbadessa, N. Gebbia, M. Tolomeo, Identification of a Terphenyl Derivative that Blocks the Cell Cycle in the G_0–G_1 Phase and Induces Differentiation in Leukemia Cells, J. Med. Chem. 49 (2006) 3012–3018. https://doi.org/10.1021/jm060253o.

[25] A. Gandini, M. Bartolini, D. Tedesco, L. Martinez-Gonzalez, C. Roca, N.E. Campillo, J. Zaldivar-Diez, C. Perez, G. Zuccheri, A. Miti, A. Feoli, S. Castellano, S. Petralla, B. Monti, M. Rossi, F. Moda, G. Legname, A. Martinez, M.L. Bolognesi, Tau-Centric Multitarget Approach for Alzheimer's Disease: Development of First-in-Class Dual Glycogen Synthase Kinase 3β and Tau-Aggregation Inhibitors, J. Med. Chem. 61 (2018) 7640–7656. https://doi.org/10.1021/acs.jmedchem.8b00610.

[26] M.L. Bolognesi, H.N. Ai Tran, M. Staderini, A. Monaco, A. López-Cobeñas, S. Bongarzone, X. Biarnés, P. López-Alvarado, N. Cabezas, M. Caramelli, P. Carloni, J.C. Menéndez, G. Legname, Discovery of a Class of Diketopiperazines as Antiprion Compounds, ChemMedChem. 5 (2010) 1324–1334. https://doi.org/10.1002/cmdc.201000133.

[27] E. Uliassi, G. Fiorani, R.L. Krauth-Siegel, C. Bergamini, R. Fato, G. Bianchini, J. Carlos Menéndez, M.T. Molina, E. López-Montero, F. Falchi, A. Cavalli, S. Gul, M. Kuzikov, B. Ellinger, G. Witt, C.B. Moraes, L.H. Freitas-Junior, C. Borsari, M.P. Costi, M.L. Bolognesi, Crassiflorone derivatives that inhibit Trypanosoma brucei glyceraldehyde-3-phosphate dehydrogenase (Tb GAPDH) and Trypanosoma cruzi trypanothione reductase (Tc TR) and display trypanocidal activity, European Journal of Medicinal Chemistry. 141 (2017) 138–148. https://doi.org/10.1016/j.ejmech.2017.10.005.

[28] A. Cavalli, M.L. Bolognesi, S. Capsoni, V. Andrisano, M. Bartolini, E. Margotti, A. Cattaneo, M. Recanatini, C. Melchiorre, A Small Molecule Targeting the Multifactorial Nature of Alzheimer's Disease, Angew. Chem. Int. Ed. 46 (2007) 3689–3692. https://doi.org/10.1002/anie.200700256.

[29] S. Bongarzone, H.N.A. Tran, A. Cavalli, M. Roberti, P. Carloni, G. Legname, M.L. Bolognesi, Parallel Synthesis, Evaluation, and Preliminary Structure−Activity Relationship of 2,5-Diamino-1,4-benzoquinones as a Novel Class of Bivalent Anti-Prion Compound, J. Med. Chem. 53 (2010) 8197–8201. https://doi.org/10.1021/jm100882t.

[30] M.L. Bolognesi, A. Cavalli, L. Valgimigli, M. Bartolini, M. Rosini, V. Andrisano, M. Recanatini, C. Melchiorre, Multi-Target-Directed Drug Design Strategy: From a Dual Binding Site Acetylcholinesterase Inhibitor to a Trifunctional Compound against Alzheimer's Disease, J. Med. Chem. 50 (2007) 6446–6449. https://doi.org/10.1021/jm701225u.

[31] P. Bey, F.N. Bolkenius, N. Seiler, P. Casara, N-(2,3-Butadienyl)-1,4-butanediamine derivatives: potent irreversible inactivators of mammalian polyamine oxidase, J. Med. Chem. 28 (1985) 1–2. https://doi.org/10.1021/jm00379a001.

[32] M.L. Bolognesi, G. Marucci, P. Angeli, M. Buccioni, A. Minarini, M. Rosini, V. Tumiatti, C. Melchiorre, Analogues of Prazosin That Bear a Benextramine-Related Polyamine Backbone Exhibit Different Antagonism toward α_1 -Adrenoreceptor Subtypes, J. Med. Chem. 44 (2001) 362–371. https://doi.org/10.1021/jm000995w.

[33] M.L. Bolognesi, R. Budriesi, A. Chiarini, E. Poggesi, A. Leonardi, C. Melchiorre, Design, Synthesis, and Biological Activity of Prazosin-Related Antagonists. Role of the Piperazine and Furan Units of Prazosin on the Selectivity for α_1 -Adrenoreceptor Subtypes, J. Med. Chem. 41 (1998) 4844–4853. https://doi.org/10.1021/jm9810654.

[34] I. Sieveking, P. Thomas, J.C. Estévez, N. Quiñones, M.A. Cuéllar, J. Villena, C. Espinosa-Bustos, A. Fierro, R.A. Tapia, J.D. Maya, R. López-Muñoz, B.K. Cassels, R.J. Estévez, C.O. Salas, 2-Phenylaminonaphthoquinones and related compounds: Synthesis, trypanocidal and cytotoxic activities, Bioorganic & Medicinal Chemistry. 22 (2014) 4609–4620. https://doi.org/10.1016/j.bmc.2014.07.030.

[35] G. Tarzia, A. Duranti, G. Gatti, G. Piersanti, A. Tontini, S. Rivara, A. Lodola, P.V. Plazzi, M. Mor, S. Kathuria, D. Piomelli, Synthesis and Structure-Activity Relationships of FAAH Inhibitors: Cyclohexylcarbamic Acid Biphenyl Esters with Chemical Modulation at the Proximal Phenyl Ring, ChemMedChem. 1 (2006) 130–139. https://doi.org/10.1002/cmdc.200500017.

[36] A.R. King, E.Y. Dotsey, A. Lodola, K.M. Jung, A. Ghomian, Y. Qiu, J. Fu, M. Mor, D. Piomelli, Discovery of Potent and Reversible Monoacylglycerol Lipase Inhibitors, Chemistry & Biology. 16 (2009) 1045–1052. https://doi.org/10.1016/j.chembiol.2009.09.012.

[37] S. Rivara, D. Pala, A. Lodola, M. Mor, V. Lucini, S. Dugnani, F. Scaglione, A. Bedini, S. Lucarini, G. Tarzia, G. Spadoni, MT_1 -Selective Melatonin Receptor Ligands: Synthesis, Pharmacological Evaluation, and Molecular Dynamics Investigation of N -{[(3- O -Substituted)anilino]alkyl}amides, ChemMedChem. 7 (2012) 1954–1964. https://doi.org/10.1002/cmdc.201200303.

[38] S. Rivara, A. Lodola, M. Mor, A. Bedini, G. Spadoni, V. Lucini, M. Pannacci, F. Fraschini, F. Scaglione, R.O. Sanchez, G. Gobbi, G. Tarzia, N -(Substituted-anilinoethyl)amides: Design, Synthesis, and Pharmacological Characterization of a New Class of Melatonin Receptor Ligands, J. Med. Chem. 50 (2007) 6618–6626. https://doi.org/10.1021/jm700957j.

[39] C. Carmi, E. Galvani, F. Vacondio, S. Rivara, A. Lodola, S. Russo, S. Aiello, F. Bordi, G. Costantino, A. Cavazzoni, R.R. Alfieri, A. Ardizzoni, P.G. Petronini, M. Mor, Irreversible Inhibition of Epidermal Growth Factor Receptor Activity by 3-Aminopropanamides, J. Med. Chem. 55 (2012) 2251–2264. https://doi.org/10.1021/jm201507x.

[40] A. Duranti, A. Tontini, F. Antonietti, F. Vacondio, A. Fioni, C. Silva, A. Lodola, S. Rivara, C. Solorzano, D. Piomelli, G. Tarzia, M. Mor, N -(2-Oxo-3-oxetanyl)carbamic Acid Esters as N -Acylethanolamine Acid Amidase Inhibitors: Synthesis and Structure–Activity and Structure–Property Relationships, J. Med. Chem. 55 (2012) 4824–4836. https://doi.org/10.1021/jm300349j.

[41] R. Castelli, M. Tognolini, F. Vacondio, M. Incerti, D. Pala, D. Callegari, S. Bertoni, C. Giorgio, I. Hassan-Mohamed, I. Zanotti, A. Bugatti, M. Rusnati, C. Festuccia, S. Rivara, E. Barocelli, M. Mor, A. Lodola, Δ5-Cholenoyl-amino acids as selective and orally available antagonists of the Eph–ephrin system, European Journal of Medicinal Chemistry. 103 (2015) 312–324. https://doi.org/10.1016/j.ejmech.2015.08.048.

[42] A. Schwartz, J.C. Allen, S. Harigaya, Possible involvement of cardiac Na^+, K^+-adenosine triphosphatase in the mechanism of action of cardiac glycosides, J Pharmacol Exp Ther. 168 (1969) 31–41.

[43] I. Hassan-Mohamed, C. Giorgio, M. Incerti, S. Russo, D. Pala, E.B. Pasquale, I. Zanotti, P. Vicini, E. Barocelli, S. Rivara, M. Mor, A. Lodola, M. Tognolini, UniPR129 is a competitive small molecule Eph-ephrin antagonist blocking in vitro angiogenesis at low micromolar concentrations: Eph antagonist and angiogenesis, Br J Pharmacol. 171 (2014) 5195–5208. https://doi.org/10.1111/bph.12669.
